# Risk factors for emergency presentation with lung and colorectal cancers: a systematic review

**DOI:** 10.1136/bmjopen-2014-006965

**Published:** 2015-04-02

**Authors:** Elizabeth D Mitchell, Benjamin Pickwell-Smith, Una Macleod

**Affiliations:** 1 Centre for Health Services Research, Leeds Institute of Health Sciences, University of Leeds, Leeds, UK; 2 Acute General Medicine, John Radcliffe Hospital, Oxford University Hospitals, Oxford, UK; 3 Supportive Care, Early Diagnosis and Advanced disease (SEDA) Research Group, Centre for Health and Population Sciences, Hull York Medical School, University of Hull, Hull, UK

**Keywords:** Early diagnosis, Lung cancer, Colorectal cancer, PRIMARY CARE

## Abstract

**Objective:**

To identify patient and practitioner factors that influence cancer diagnosis via emergency presentation (EP).

**Design:**

Systematic review.

**Data sources:**

MEDLINE, EMBASE, CINAHL, EBM Reviews, Science and Social Sciences Citation Indexes, Conference Proceedings Citation Index-Science and Conference Proceedings Citation Index-Social Science and Humanities. Searches were undertaken from 1996 to 2014. No language restrictions were applied.

**Study selection:**

Studies of any design assessing factors associated with diagnosis of colorectal or lung cancer via EP, or describing an intervention to impact on EP, were included. Studies involving previously diagnosed cancer patients, assessing only referral pathway effectiveness, outcomes related to diagnosis or post-EP management were excluded. The population was individual or groups of adult patients or primary care practitioners. Two authors independently screened studies for inclusion.

**Results:**

22 studies with over 200 000 EPs were included, most providing strong evidence. Five were graded ‘insufficient’, primarily due to missing information rather than methodological weakness. Older patient age was associated with EP for lung and colorectal cancers (OR 1.11–11.03 and 1.19–5.85, respectively). Women were more at risk of EP for lung but not colorectal cancer. Higher deprivation increased the likelihood of lung cancer EP, but evidence for colorectal was less conclusive. Being unmarried (or divorced/widowed) increased the likelihood of EP for colorectal cancer, which was also associated with pain, obstruction and weight loss. Lack of a regular source of primary care, and lower primary care use were positively associated with EP. Only three studies considered practitioner factors, two involving diagnostic tests. No conclusive evidence was found.

**Conclusions:**

Patient-related factors, such as age, gender and deprivation, increase the likelihood of cancer being diagnosed as the result of an EP, while cancer symptoms and patterns of healthcare utilisation are also relevant. Further work is needed to understand the context in which risk factors for EP exist and influence help-seeking.

**Strengths and limitations of this study**

- This review has for the first time synthesised available evidence on factors associated with diagnosis of colorectal or lung cancer during an emergency presentation. As such, it is a valuable addition to previous work demonstrating that patients with cancer diagnosed in this way have poorer outcomes.
- While we carried out a comprehensive review of the world literature, few evaluative studies were identified, with most researchers undertaking observational work utilising routine data.
- Variations in study design, populations under study and the healthcare settings involved precluded pooling data for meta-analysis. Nevertheless, while the majority of studies were descriptive in nature, they were for the most part of good quality.
- Despite the lack of controlled studies, we have been able to carry out a definitive synthesis of existing evidence on this topic, and have identified some clear messages.
- Evidence from the review demonstrates that patient-related factors, such as age, gender and socioeconomic deprivation, increase the likelihood of cancer being diagnosed as the result of an emergency presentation, and that cancer symptoms and patterns of healthcare utilisation are also relevant.

## Introduction

Cancer remains one of the UK's biggest health issues, both in terms of morbidity and mortality. In recent years, there has been increasing interest in the pathway to diagnosis,^1^ as international data have shown that 1 year survival for many cancers is poorer in the UK than in comparable countries.^2^ ^3^ As such, it is important to target groups where some of the worst outcomes have been identified, as there is potentially most to gain from understanding pathways to diagnosis for such patients.

In the UK, the main routes to cancer diagnosis are considered to be screen detected, 2-week wait (2WW), general practitioner (GP) referral (not under 2WW), referral from another hospital specialty and emergency presentation (EP). The 2WW system (introduced in 2000), whereby patients are referred urgently for suspected cancer by their GP and can expect to be seen by a specialist within 2 weeks, is seen as being the gold standard for cancer diagnosis.^4^ Conversely, it is well established that for the majority of cancers, diagnosis within the context of an EP results in poorer outcomes.^5^ Between 2006 and 2010, 23% of all cancers in England were diagnosed via the emergency route, with even greater proportions of lung (38%) and colorectal (25%) cancers diagnosed in this way (http://www.ncin.org.uk/publications/routes_to_diagnosis). Across all cancer sites, 1 year relative survival was significantly lower than for presentations by other routes, and was considerably lower than for diagnoses made via the 2WW pathway (colorectal: all routes 74%, 2WW 83%, emergency 49%; lung: all routes 29%, 2WW 42%, emergency 11%).

Despite the undoubted benefits of improving the diagnostic pathway for these patients, as yet we know little about the context surrounding the route to EP. The majority of published work has focused on outcomes,^6^ ^7^ and as such, there is a dearth of evidence relating to the factors that influence the presentation itself. The purpose of this review was to identify the patient and practitioner factors that influence cancer diagnosis via EP, using lung and colorectal cancers as exemplars, and to determine whether any interventions have been found to impact on EP.

## Methods

### Terminology

The term ‘emergency presentation’ in the cancer literature is used to denote a variety of routes to diagnosis. For the purposes of this review, EP was defined as *a diagnosis of cancer that arose during an unscheduled (or emergency or unplanned) hospital admission*, whether that admission was initiated by the patient seeking management of the condition through an emergency portal (such as the emergency department (ED)), by a primary healthcare practitioner (including out of hours) admitting a patient to the ED, acute medical or surgical unit for management of an illness that is subsequently diagnosed as cancer during that admission, or by a hospital practitioner admitting a patient directly from an outpatient clinic.

### Identification of studies

A search of the world literature from 1996 to March 2014 was undertaken. This timescale was selected as it covers the period following the introduction of the Calman-Hine report (1995), which set out a strategic framework for creating a network of cancer care in England and Wales, thereby transforming cancer services. We searched MEDLINE, Ovid (1996 to February week 2 2014), EMBASE, Ovid (1996 to 2014 week 9), CINAHL, Ebsco (1996 to March 2014), EBM Reviews (including the Cochrane Database of Systematic Reviews), Ovid (1996 to January 2014), Science Citation Index, ISI Web of Science (1996 to March 2014) and Social Sciences Citation Index, ISI Web of Science (1996 to March 2014). Supplementary searches of Conference Proceedings Citation Index-Science and Conference Proceedings Citation Index-Social Science and Humanities, ISI Web of Science (1996 to March 2014) were conducted to provide relevant unpublished work. In addition, the reference lists of included studies were reviewed for potentially relevant papers. A range of MeSH headings and keyword searches were used including Primary Health Care/, Secondary Care/, “emergency”, “unscheduled”, “admission”, and “present*”. A sample search is provided in online supplementary appendix 1.

### Criteria for inclusion

Studies were included if the participants were individual or groups of adult patients or primary care practitioners, and they considered factors that were associated with diagnosis of colorectal or lung cancer in the context of an EP, or they described an intervention designed to impact on emergency cancer presentation. Studies of any research design were considered (with the exception of single case reports), and no language restrictions were imposed. Studies involving patients who had previously had a diagnosis of cancer were excluded, as were those that assessed only the effectiveness of specific referral pathways (eg, 2WW). Similarly, studies focusing only on outcomes related to diagnosis via EP or on management following EP were also excluded (see online supplementary appendix 2).

### Screening and data extraction

Titles and abstracts of all identified studies were independently screened for eligibility by two reviewers, and full-text versions of papers not excluded at this stage obtained for detailed review. Potentially relevant studies were then independently assessed to determine if they met the inclusion criteria. Differences of opinion were discussed until a consensus was reached, with the opinion of a third reviewer sought where necessary. Data extraction on a sample of included studies (n=10) was carried out by two reviewers (BP-S and EDM) using a standardised proforma, with the remainder completed by one reviewer (EDM). Data included research design and location, study setting, participants, emergency pathway, and results.

### Assessment of evidence

Assessment of study evidence was carried out by one reviewer (EDM). Where possible, studies were evaluated using previously developed scoring systems (ie, the Newcastle-Ottawa scale for case–control studies). However, many of the papers included in this review used methodologies that did not lend themselves to the scoring systems outlined. A method of assessing the strength of evidence of observational studies—developed as part of a previous systematic review on early diagnosis of cancer^8^ ^9^—was therefore modified for this topic area, and applied to relevant studies.

In this system, papers were evaluated on the basis of ‘population’, ‘ascertainment’ and ‘analysis’ (see online supplementary appendix 3). Population relates to the method used to ensure an appropriately powered study/generalisable results, with use of a sample size calculation or inclusion of all possible patients/providers rated more highly than selective recruitment. Ascertainment relates to methods of obtaining study data, with use of a rigorous method designed to reduce systematic differences between groups (selection, characteristics, etc) rated more highly than other methods. Finally, analysis relates to use of analytic techniques, with reporting of relevant statistical comparisons/differences (or use of appropriate analytic techniques if qualitative) rated more than highly than non-statistical comparisons or descriptive data. Evidence was assessed as *strong* if a paper was graded strong for population, ascertainment and analysis, *strong–* if graded as strong for two of the areas and moderate for one area, and *moderate* if graded as strong for one area and moderate for two areas, or as moderate for all three areas. Evidence was considered to be *insufficient* if a paper used a selective study population and/or an inappropriate method to ascertain data, or if it did not provide enough information to be able to determine a grading. Studies of low quality were not excluded from the review, but were interpreted in light of this.

### Data synthesis

Substantial clinical and methodological heterogeneity between studies, along with a dearth of controlled comparisons, meant that it was not appropriate to pool data for meta-analysis. Instead, we carried out a narrative synthesis of findings to identify key concepts and themes relating to EP that were shared across individual studies. In addition, the evidence generated by each study has been assessed on the robustness of its methodology and analysis, allowing us to weight each study in our composite assessment of risk factors for EP.

## Results

### Description of studies

#### Search results

**Figure 1:**
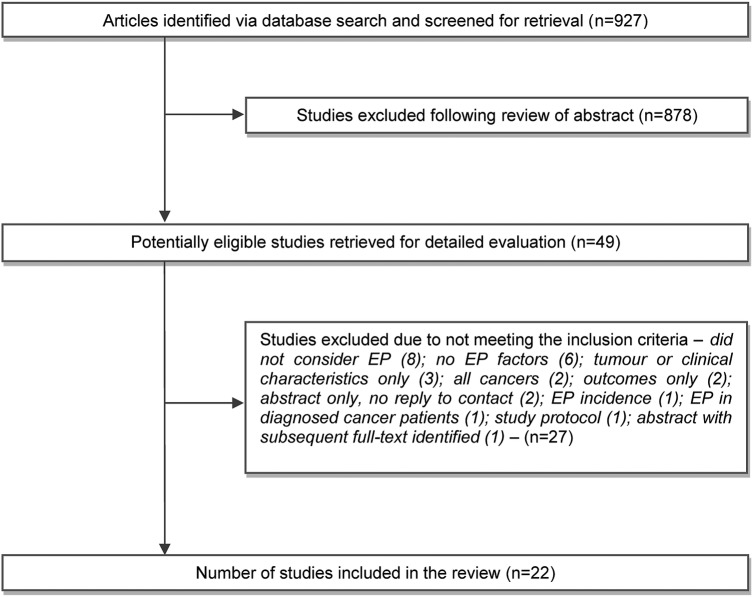
Flow of studies into the review.

The search strategy identified 927 articles of which 49 appeared to meet the inclusion criteria and were subject to detailed review (figure 1). Twenty-two papers, involving more than 687 000 individuals with lung or colorectal cancer, were included in the final analysis (tables 1–3), including five studies identified from the grey literature. No non-English language studies were included, and no relevant qualitative work was identified. Three papers dealt with lung cancer alone, 16 dealt with colorectal cancer (1 also involving upper gastrointestinal cancer), and 3 considered both lung and colorectal cancers (plus breast cancer in 2 cases). The majority dealt with factors relevant to patients; only three reported on practice-related issues. Only one study (before and after design) evaluated an intervention.

**Table 1:** Patient-based factors for EP with lung cancer

Authors	Design	Patient group	Comparator(s)	Associated	Not associated	Evidence
Beckett *et al* (UK, England; 2014)^11^	Observational (retrospective analysis of cancer audit data)	25 675 EP patients (median age 74); 56% male	99 522 elective referrals (median age 72); 57% male	Age—older (80 to >90 years, OR 1.11–1.68); deprivation—higher (OR 0.92, least deprived); performance status—poorer (PS 1–4, OR 1.58–7.28)	Comorbidity	S
[Linek *et al*] (USA; 2011)^23^	Cross-sectional	322 patients		Ethnicity—black, Hispanic (p=0.01)		I
Melling *et al* (UK, England; 2002)^28^	Observational (retrospective analysis of cancer registry data, and medical records review)	41 EP patients (27% aged >75); 59% male	173 elective referrals with CXR (25% aged >75); 61% male 148 elective referrals without CXR (39% aged >75); 66% male	Gender—female (41.5% vs 39.3% vs 34.5%); symptoms—non-respiratory (respiratory 39% vs 86% vs 52%)	Age	S–
Pollock and Vickers (UK, England; 1998)^16^	Observational (retrospective analysis of HES and census data)	38 668 patients		Deprivation—higher (Q2-Q10, OR 1.21–2.20)		S
Raine *et al* (UK, England; 2010)^18^	Observational (retrospective analysis of HES data)	96 521 EP admissions (aged 50 to 90+); 59% male	90 220 non-EP admissions (aged 50 to 90+); 62% male	Age—older (60 to ≥90 years, OR 1.23–11.03); gender—female (OR 1.12); deprivation—higher (OR 0.64, least deprived)		S
Sikka and Ornato (USA; 2012)^19^	Observational (retrospective analysis of cancer registry, health insurance and census data)	2186 EP patients (28% aged >80); 55% male	9095 non-emergency patients (22% aged >80); 57% male	Age—older (80 to ≥85 years, OR 1.33–1.52); gender—female (OR 1.13); ethnicity—African-American (OR 1.42); comorbidity (score 1 to 3+, OR 3.79–12.44); primary care use—lower (≥1 visit, OR 0.58); secondary care use—higher (≥1 admit, OR 1.21); ED use—higher (p<0.05)	Annual household income; Medicaid, Medicare insurance	S

[Abstract only].

CXR, chest X-ray; EP, emergency presentation; HES, Hospital Episode Statistics; I, insufficient; M, moderate; S–, strong–; S, strong.

**Table 2:** Patient-based factors for EP with colorectal cancer

Authors	Study design	Patient group	Comparator(s)	Associated	Not associated	Evidence
[Askari *et al*] (UK, England; 2013)^10^	Observational (retrospective analysis of hospital colorectal cancer database)	237 emergency surgeries	1025 elective surgeries	Cancer site—colon (OR=2.76–4.83)	Age; gender; deprivation; ethnicity; comorbidity	M
Cleary *et al* (UK, England; 2007)^27^	Case–control	62 EP patients (median age 75); 44% male	287 elective patients (median age 73); 52% male 310 age-sex matched controls	Symptoms—abdominal pain (OR=6.2), weight loss (OR=3.4), diarrhoea (OR=2.6)	Symptoms—rectal bleeding	S–
[Gould *et al*] (Australia; 2013)^29^	Observational (retrospective analysis of admission episodes)	30 EP patients (mean age 75)		Symptoms—bowel obstruction (±perforation)		I
Gunnarsson *et al* (Sweden; 2011)^21^	Case–control (nested)	97 EP patients (median age 77); 49% male	488 elective patients (median age 74); 50% male	Age—older (median 77 vs 74 years, p=0.02); cancer site—ascending/sigmoid colon (p=0.04)	Gender; comorbidity (hypertension); marital status; home ownership; residence (urban/rural)	M
Gunnarsson *et al* (Sweden; 2013)^12^	Observational (retrospective analysis of cancer registry, labour and tax data)	2856 EP patients (35% aged >80); 47% male	9437 elective patients (28% aged >80); 49% male	Age—older (≥80 years p<0.001*); marital status—unmarried (OR=1.24); income—lower (OR=1.22); education—lower (p=0.018*); childlessness (p=0.021*)	Gender	S
[Khamizar *et al*] (Malaysia; 2010)^30^	Observational (retrospective analysis of cancer registry data)	42 emergency surgeries	123 elective surgeries	Symptoms—abdominal pain, change in bowel habit; cancer site—colon		I
Khattak *et al* (UK, England; 2006)^22^	Cross-sectional	58 emergency admissions (median age 72); 57% male (of all patients)	43 elective admissions (median age 72); 57% male (of all patients)	Symptoms—shorter time to presentation (median 11.5 vs 49.5 days, p=0.04)		M
MacDonald *et al* (UK, Scotland; 2011)^13^	Observational (retrospective analysis of cancer audit data)	395 EP patients (mean age 71); 56% male (of all patients)	1223 elective patients (mean age 68); 56% male (of all patients)	Age—older (mean 70.6 vs 67.9 years p<0.005)		S
Mitchell *et al* (Canada; 2007)^24^	Cross-sectional	108 emergency resections (mean age 71); 40% male	347 elective resections (mean age 67); 56% male	Age—older (mean 70.8 vs 67.0 years, p=0.005); Gender—female (29.7% vs 18.2%, p=0.004); symptoms—obstruction (43% vs 2%), pain (23% vs 8%); BMI—extreme (<25 or >40, p=0.001)	Annual household income; Education level; smoking history; family history; previous CRC screening	S−
Oldale *et al* (UK, England; 2000)^25^	Cross-sectional	100 emergency admissions (50% aged ≥75); 47% male	357 elective admissions (37% aged ≥75); 59% male	Age—older (≥75 years, 42% vs 34%, p=0.05); marital status—single, divorced, widowed (44% vs 35%, p=0.07)	Gender; deprivation	S
Oliphant *et al* (UK, Scotland; 2013)^14^	Observational (retrospective analysis of cancer registry, SMR1 and deaths data)	945 EP patients	3351 elective patients	Deprivation—higher (23.5% vs 19.5%, p=0.033)		S
Polednak (USA; 2000)^15^	Observational (retrospective analysis of cancer registry and hospital discharge data)	2183 emergency admissions (56% aged ≥75); 46% male	8840 all other route patients (39% aged ≥75); 51% male	Age—older (75 to ≥85 years, OR=1.89–3.42); Gender—female (53.8% vs 48.7%, p<0.001*); ethnicity—black (OR=1.76); insurance—self-pay (OR=2.08), Medicaid (OR=2.66)		S
Pollock and Vickers (UK, England; 1998)^16^	Observational (retrospective analysis of HES and census data)	53 742 patients		Deprivation—higher (Q3-Q10 OR=1.27–2.29)		S
Porta *et al* (Spain; 1998)^26^	Cross-sectional	161 emergency admissions (median age 67); 63% male	87 elective admissions (median age 70); 64% male	Symptoms—anorexia, weakness, weight loss (24.8% vs 9.2%, p<0.01); symptoms—no presentation at first symptom (34.2% vs 13.8%, p<0.01)	Age; gender; social class; family history; cancer site	S–
Rabeneck *et al* (Canada; 2006)^17^	Observational (retrospective analysis of Institute for Health information and health insurance data)	7739 OPE patients (64% aged >70); 50% male	33 617 non-OPE patients (53% aged >70); 54% male	Age—older (10-year increment OR=1.19); gender—female (male, OR=0.93); income—lower (highest income, OR=0.78); comorbidity (score 1 to ≥3, OR=1.80–3.51); primary care use—lower (regular care, OR=0.70); previous investigation—lower (Ix in 5 years, OR=0.69)		S
Raine *et al* (UK, England; 2010)^18^	Observational (retrospective analysis of HES data)	60 684 EP admissions (aged 50–90+); 51% male	126 293 non-EP admissions (aged 50–90+); 57% male	Age—older (70 to ≥90years, OR=1.41–5.85); gender—female (OR=1.15); deprivation—higher (OR=0.66, least deprived)		S
Sikka and Ornato (USA; 2012)^19^	Observational (retrospective analysis of cancer registry, health insurance and census data)	2092 EP patients (43% aged >80); 42% male	6938 non-emergency patients (33% aged >80); 47% male	Age—older (75 to ≥85 years, OR=1.45–1.89); gender—female (OR=1.18); health insurance (OR=1.37); comorbidity (score 1 to 3+, OR=1.89–4.11); primary care use—lower (≥1 visit, OR=0.68); secondary care use—higher (≥1 admit, OR=1.29); ED use—higher (p<0.05)	Annual household income; ethnicity	S
[Sivakumaran *et al*] (Australia; 2013)^31^	Observational (retrospective medical records review)	97 EP patients (median age 76); 56% male (of all patients)	223 elective patients (median age 69); 56% male (of all patients)	Age—older (median 76 vs 69 years, p<0.001); cancer site—colon (rectal cancer, OR=3.20)		I

[Abstract only].

*Associated in univariate analysis.

BMI, body mass index; CRC, colorectal cancer; EP, emergency presentation; HES, Hospital Episodes Statistics; I, insufficient; Ix, investigation; M, moderate; S–, strong–; S, strong; SMR, Scottish Morbidity Recording.

**Table 3:** Practitioner-based factors for EP with lung or colorectal cancer

Authors	Study design	Cancer	Patient group	Comparator(s)	Associated	Not associated	Evidence
Melling *et al* (UK, England; 2002)^28^	Observational (retrospective analysis of cancer registry data, and medical records review)	Lung	41 EP patients (27% aged >75); 59% male	173 elective referrals with CXR (25% aged >75); 61% male 148 elective referrals without CXR (39% aged >75); 66% male	Pathway—no CXR (inferred)		S–
Davies *et al* (UK, England; 2004)^20^	Before and after	CRC	84 preflexible sigmoidoscopy patients (30 EP); 51% male	635 postflexible sigmoidoscopy patients (165 EP); 51% male	Pathway—use of a fast-track flexible sigmoidoscopy referral system (EP fell from 35.7% to 25.9%, p=0.059)		I
Oldale *et al* (UK, England; 2000)^25^	Cross-sectional	CRC	100 emergency admissions (50% aged ≥75); 47% male	357 elective admissions (37% aged ≥75); 59% male		Practice size (GPs); fundholding status; training status	S

CRC, colorectal cancer; CXR, chest X-ray; EP, emergency presentation; GP, general practitioner; I, insufficient; S–, strong–; S, strong.

#### Populations

Two-thirds of the studies were carried out in Europe (n=14; 64%), most in the UK (n=11). The remainder were sited in North America (n=5; 23%), Australia (n=2; 9%) and Malaysia (n=1; 4%). Studies were relatively large in size, involving between 30 and 373 718 participants (mean 31 269; median 689). In total, more than 200 000 EPs (mean 10 125; median 178) were included. It was not possible to determine actual numbers of EPs for two studies, one abstract only inclusion and one paper where a complete breakdown of numbers was not provided.

In the main, studies with the largest number of participants (>1000) analysed data for cases identified from routine data sources, collected either at national (Hospital Episodes Statistics, National Lung Cancer Audit), regional (state-wide cancer registry, state-wide discharge data, Managed Clinical Network, health authority data) or local level (hospital cancer database).^10–19^ In the remaining studies (n=12), more than half involved patients admitted to hospital (62%).^20–26^ Only one study was based in primary care.^27^ All but one of the studies considered patient-related factors, but only four included patients directly as participants; none included practitioners.

#### Emergency presentations

In the majority of studies (n=15; 68%), the authors included a definition of the term ‘emergency presentation’. For the most part, EP related to an admission made at short notice (either as a result of referral from a GP or other practitioner, or because the patient attended the ED) or to a diagnosis made in the ED or inpatient setting.^11^ ^15^ ^16^ ^19^ ^23–26^ ^28^ In some of the colorectal cancer literature however, the definition went beyond admission to include surgery (sometimes within a given timeframe) and/or a specific presenting symptom such as obstruction or perforation.^12–14^ ^20^ ^21^ ^27^

### Quality assessment

None of the included studies employed a controlled trial methodology, with most (n=15; 68%) involving secondary analysis of routine data, with or without complementary medical records review.^10–19^ ^21^ ^27–30^ Only six studies collected data prospectively, four using a combination of administered patient questionnaire and medical records review.^20^ ^22–26^ Fourteen papers were assessed as providing strong evidence (four graded as strong–), three provided moderate evidence and five were insufficient. Four of the insufficient papers were abstract only inclusions that provided limited information,^23^ ^29–31^ while the final paper lacked detail on identification of patients and methods for collecting data.^20^

### Patient-related risk factors for EP

#### Demography

Demographic characteristics were the most commonly evaluated factors related to EP, and were considered in 17 of the 22 included papers (tables 1, 2 and 4).

**Table 4:** Risk factors for emergency cancer presentation (number of studies)

Risk factor	Colorectal cancer												Lung cancer			
Demographic																
Age (older)	●	●	●	●	●	●	●	●	●	⊙	○	○	●	●	●	○
Gender (female)	●	●	●	●	●	○	○	○	○	○			●	●	●	
Deprivation (higher)	●	●	●	○	○								●	●	●	
Annual income—household, individual (lower)	●	●	○	○									○			
Ethnicity (non-white origin)	●	○	○										●	⊙		
Enrolment in health insurance	●	●											○			
Marital status (unmarried, divorced, widowed)	●	●	○													
Education level (lower)	●	○														
Social class (lower)	○															
Residence (ownership, location)	○															
Childlessness	●															
History																
Cancer site (colon)	●	●	⊙	⊙	○											
Symptom type	●	●	●	⊙	⊙								●			
Symptom type (pain)	●	●	⊙													
Symptom type (weight loss)	●	●														
Symptom type (obstruction)	●	⊙														
Symptom type (change in bowel habit)	●	⊙														
Symptom type (bleeding)	○															
Help-seeking at initial symptom	●	○														
Comorbidity	●	●	○	○									●	○		
Performance status (poorer)													●			
Smoking history	○															
BMI (extreme)	●															
Primary care utilisation (lower)	●	●											●			
Secondary care utilisation (higher)	●	○											●			
Previous screening/investigation	●	○														
Family history of cancer	○	○														

● Study reports association with EP (evidence rated as ‘strong’, ‘strong−’ or ‘moderate’); ⊙ study reports association with EP (evidence rated as ‘insufficient’); ○ study reports no association with EP.

BMI, body mass index; EP, emergency presentation.

Older patient age was found to be a significant factor in all but three of the 14 studies evaluating this, and was associated with EP for both lung and colorectal cancers.^10–13^ ^15^ ^17–19^ ^21^ ^24–26^ ^28^ ^31^ While the specific at-risk age varied across studies (60 to ≥90 years), those using multivariate regression analysis found the odds of EP to be between 1.11 and 11.03 for older patients with lung cancer, and between 1.19 and 5.85 for older patients with colorectal cancer. Women were found to be more at risk of EP for lung cancer,^18^ ^19^ ^28^ but there was no clear evidence that this was the case for colorectal cancer, with studies split between those finding a positive association^15^ ^17–19^ ^24^ and those finding none.^10^ ^12^ ^21^ ^25^ ^26^

Higher socioeconomic deprivation increased the likelihood of EP for lung cancer,^11^ ^16^ ^18^ but the evidence of a relationship with presentation for colorectal cancer was less conclusive.^10^ ^14^ ^16^ ^18^ ^25^ Those studies that did identify an association found that between 4% and 21% more patients in the most deprived group presented as emergencies compared with patients in the most affluent group. While there was little evidence of an association between annual household income and EP in either cancer group,^12^ ^19^ ^24^ one study found that lower individual patient income was linked to emergency diagnosis of colorectal cancer.^17^ Two studies from the USA found that enrolment in the Medicaid insurance scheme (OR=2.66; 95% CI 1.89 to 3.72), or self-paying medical fees (OR=2.08; 95% CI 1.30 to 3.33) increased the likelihood of EP for colorectal cancer.^15^ ^19^ However, this was not the case for lung cancer.^19^ There was also some evidence that non-white ethnic origin was a risk factor for EP, although in the case of colorectal cancer this was inconclusive.^10^ ^15^ ^19^

Several additional factors were studied in relation to EP with colorectal cancer. No definitive relationship between education level,^12^ ^24^ residence (either geographical area or home ownership)^21^ and social class^26^ was identified, but there was evidence to suggest that being unmarried (and in some cases divorced or widowed) increased the likelihood of EP.^12^ ^21^ ^25^ In addition, one study found that the risk of EP was increased in those with no children (p=0.021), although this was not statistically significant in multivariate analysis.^12^

#### History

In some cases, the type of symptoms that patients experienced had an effect on presenting behaviour (tables 1, 2 and 4). One lung cancer study found that patients with non-respiratory symptoms were more likely to present as an emergency than patients with lung-related symptoms (cough, chest pain or infection, haemoptysis or dyspnoea), who were more likely to attend their GP and be referred electively with a chest X-ray already carried out (39% EP vs 80% elective).^28^ Similarly, EP for colorectal cancer was found to be linked to more serious symptoms, such as pain, obstruction and weight loss.^24^ ^26^ ^27^ ^29^ ^30^ There was also some evidence to suggest that patients with colon cancer were more likely to present as emergencies than those with rectal cancer.^10^ ^21^ ^26^ ^30^ ^31^ Perhaps unsurprisingly then, rectal bleeding was not found to be associated with EP, possibly as a result of patients seeking help earlier with what they might consider to be a more alarming symptom.^27^ One study found that the first cancer symptom triggered help-seeking less often in emergency patients (65.8% vs 86.2%; p<0.01),^26^ while another found that the time between first symptom and first presentation was lower for patients presenting as emergencies (median 11.5 vs 49.5 days; p=0.04).^22^

There was conflicting evidence about the impact of coexisting morbidity on presenting behaviour. Half of the studies evaluating this found that it increased the likelihood of EP, while the other half found that it did not; this was found to be the case for both lung^11^ ^19^ and colorectal cancers.^10^ ^17^ ^19^ ^21^ However, the method of identifying (national cancer audit, cancer registry, hospital records) and classifying comorbidity (individual conditions, Charlson score, Deyo score) varied across studies, and this may have impacted on the consistency of findings. One lung cancer study found that the odds of EP were higher for patients with worse performance status (PS4 OR=9.14; 95% CI 8.51 to 9.82),^11^ while an additional colorectal study found a significant association between body mass index (BMI) and EP, with underweight (BMI <25) and severely obese patients (BMI >40) having the highest rates of emergency diagnosis (32% and 42% of patients, respectively; p=0.001).^24^ There was no evidence of an association with smoking history.^24^

There was evidence that lack of a regular source of primary care,^17^ and lower use of primary care (no visits in the 12 months before diagnosis)^19^ were positively associated with EP, the latter for both lung and colorectal cancers. One of the studies also found that lung and colorectal patients who had at least one hospital admission in the 12 months before diagnosis were more likely to present as emergencies. In addition, patients diagnosed in the ED had a significantly higher average number of ED visits before cancer diagnosis compared with patients diagnosed in other settings (lung 0.61 vs 0.33; colorectal 0.58 vs 0.29; p<0.05).^19^ However, both studies were undertaken in North America, and given differences in healthcare provision may not be wholly generalisable to a UK setting.

Patients who had not had a bowel-related investigation in the 5 years prior to diagnosis of colorectal cancer (colonoscopy, flexible sigmoidoscopy, barium enema) were found in one study to be at increased risk of EP (OR for investigation 0.69; 95% CI 0.63 to 0.75).^17^ Conversely, previous colorectal cancer screening did not appear to have any impact, although the authors of this Canadian study did remark that there was no organised screening programme in place at that time, and only 24% of the patient cohort had ever had a screening test for colorectal cancer.^24^ There was no evidence that family history of colorectal cancer had an effect on presentation behaviour.^24^ ^26^

### Practice-related risk factors for EP

Only three studies reported on aspects of practice that could be considered to be related to EP (table 3).

In one observational before and after study, Davies *et al*^20^ studied the impact of the introduction of a fast-track flexible sigmoidoscopy referral system. They found that EPs for colorectal cancer fell from 35.7% in the year prior to the introduction of the service to 25.9% in the years following, although the difference did not reach statistical significance (p=0.059). One study from the UK found that the organisational characteristics of a patient's registered practice (number of GPs, fundholding status, training status) were not significant in relation to whether a patient was diagnosed electively or as an emergency.^25^ The study did not consider the characteristics of individual GPs which may have been more likely to impact on referral behaviours. The final study did not consider practice aspects per se, but rather inferred that the inclusion of certain components in the referral pathway would reduce EP, namely that patients with lung cancer should have a chest X-ray carried out.^22^ However, most emergency patients in the study did not present with respiratory symptoms, and as such, the indications for chest X-ray would undoubtedly be limited.

## Discussion

### Principal findings

Our review has for the first time synthesised available evidence on factors associated with diagnosis of colorectal or lung cancer during an EP. As such, it is a valuable addition to previous work that demonstrates that patients diagnosed with cancer in the context of an EP have poorer outcomes.^5^ In undertaking this review, we have also demonstrated that in general this topic is under-researched, yet despite the relative lack of evidence, the review has established a number of associations. We have identified older age in both colorectal and lung cancers as a risk factor for EP, and have shown a link between EP for lung cancer with women and more deprived groups. There is also some evidence that these factors may increase the likelihood of EP for colorectal cancer, although this is not conclusive. Other demographic factors which may be a proxy for living alone (unmarried, divorced, widowed) also appear to have an association with emergency diagnosis of colorectal cancer.

Unsurprisingly, symptoms associated with bowel obstruction (such as pain) were more likely to result in an EP for colorectal cancer, but interestingly, so too were some symptoms that are likely to have been present for longer, such as weight loss. Patients with lung cancer who did not have commonly associated respiratory symptoms that might lead to a chest X-ray (cough, chest pain, or infection) or a red flag symptom, such as haemoptysis, also had increased odds of presenting as an emergency.

Although fewer studies reported on more process-based issues, lower primary care and higher secondary care utilisation were perhaps unsurprisingly associated with diagnosis during EP. However, it was not possible to determine the reasons associated with these utilisation patterns, and as such, we are limited in what we can learn from them.

### Strengths and limitations

While we carried out a comprehensive review of the world literature, few evaluative studies were identified, with most researchers undertaking observational work utilising routine data. No randomised controlled trials were identified (perhaps as expected given the area of study), and variations in study design, populations under study and the healthcare settings involved precluded pooling data for meta-analysis. Nevertheless, while the majority of studies were descriptive in nature, they were for the most part of good quality. Only five studies were rated as insufficient, four of which were identified from the grey literature and were limited by the completeness of their reporting. Despite the lack of controlled studies, we have been able to carry out a definitive synthesis of existing evidence on this topic, and have identified some clear messages.

### Implications for clinicians, policy and research

There is a paucity of research into the factors associated with reasons for diagnosis of cancer during an EP. We were able to identify only 22 studies considering possible risk factors, and with the exception of studies reporting age and gender, we were unable to find more than five, often less, evaluating any of the other demographic or patient-related factors identified. Additionally, there is a dearth of evidence related to the potential impact of primary care practice and practitioner-related factors on EP, and limited research designed to identify and evaluate possible interventions.

While this review has shed some light on some of the factors that are associated with this, we are still unable to determine what happens to patients before they present, and indeed, whether this links to previous work on patient and practitioner delay.^8^ ^9^ It is often hypothesised that patients who present as emergencies have had some delay on the pathway to diagnosis, but as yet we are unable to conclude that this is the case. A case–control study funded by the National Awareness and Early Diagnosis Initiative (NAEDI) and starting in 2014 is designed to provide some of this evidence.^32^ It is vital that we understand this pre-presentation phase if we are to develop interventions to impact on the poor outcomes associated with diagnosis during EP. While it may be the case that not all patients with lung and colorectal cancers (especially lung cancer) would benefit from earlier diagnosis in terms of mortality, ensuring that delays in the pathway to diagnosis are minimised would at the very least be transformative to patient experience. In addition, work is needed to develop an understanding of why older people and women appear more likely to have cancers diagnosed in this way.

## Conclusions

Evidence from this review has demonstrated that certain patient-related factors, such as age, gender and socioeconomic deprivation, have an influence on diagnosis of cancer during an EP. It also shows that cancer symptoms and patterns of healthcare utilisation are relevant. While it may be the case that such patients become sick very quickly and need to be admitted to hospital, further work is needed to understand the context in which risk factors for EP exist and influence help-seeking behaviour. Until then, we may be unable to develop suitable interventions to ensure that patients are detected earlier in their pathway to diagnosis.
